# Full-Genome Sequence Analysis of a Reassortant Strain of *Bluetongue virus* Serotype 16 from Southern India

**DOI:** 10.1128/genomeA.00783-16

**Published:** 2016-08-18

**Authors:** Lalit Kumar, Kanisht Batra, Deepika Chaudhary, Akhil Kumar Gupta, Anita Dalal, Brindha Kalyanaraman, Ganesan P. Irulappan, Vinay Kumar, Sushila Maan

**Affiliations:** a College of Veterinary Sciences, LLR University of Veterinary and Animal Sciences, Hisar, Haryana, India; b Centre for Animal Health Studies, Tamil Nadu Veterinary and Animal Sciences University, Madhavaram Milk Colony, Chennai, India

## Abstract

The complete genome sequence of a reassortant field strain (IND2014/01) of *Bluetongue virus* (BTV) serotype 16, isolated from sheep from southern India in 2014, was sequenced. The total genome size was 19,186 bp. Sequence comparisons of all genome segments, except segment 5 (Seg-5), showed that IND2014/01 belonged to the major eastern topotype of BTV.

## GENOME ANNOUNCEMENT

*Bluetongue virus* (BTV) is the type species of the genus *Orbivirus* within the family *Reoviridae* (1), which causes economically important bluetongue disease in domestic and wild ruminants. BTV is a nonenveloped, icosahedral, and double-stranded RNA virus containing three concentric protein layers, transmitted biologically by certain species of *Culicoides* biting midges (2–4). The linear double-stranded RNA (dsRNA) consists of 10 segments encoding seven structural (VP1 to VP7) and five nonstructural (NS1 to NS5) proteins (5, 6). Twenty-seven serotypes of BTV have been reported so far, and there is evidence of two additional putative serotypes (5). There are also characteristic regional variants (topotypes) of each genome segment, which have developed due to separate evolution of BTV strains from different continents by acquiring multiple point mutations, insertion/deletion, and reassortment events. Complete genome data become crucial in order to explicate the emergence and molecular epidemiology of these viruses with segmented genome.

In India, 11 BTV serotypes (BTV-1, BTV-2, BTV-3, BTV-5, BTV-9, BTV-10, BTV-12, BTV-16, BTV-21, BTV-23, and BTV-24) have been isolated since 2001 (6). BTV-16 has been reported earlier from the southern states of India, and at least four complete genomes of BTV-16 have been published from India, China, Australia, and the South African reference (7–10).

We report here the whole-genome sequence analysis of an Indian isolate (IND2014/01) of BTV-16 that was isolated from sheep blood in the Karur district of Tamil Nadu. The virus was isolated using KC cells and then grown in bulk in BHK-21 cells. The viral dsRNA was purified using TRIzol reagent (Life Technologies), which was then used to synthesize cDNA by full-length amplification of cDNA (FLAC) method (11), followed by sequencing on a capillary sequencer using gene-specific primers (11).

Segments 1 to 10 (Seg-1 to Seg-10) of IND2014/01 are 3,945, 2,931, 2,772, 1,981, 1,765, 1,637, 1,156, 1,125, 1,052, and 822 bp, respectively. Phylogenetic analysis showed that IND2014/01 contains genome segments derived mainly from eastern lineages, as segments 1, 3, 7, 8, 9, and 10 have grouped within the eastern topotype along with viruses from Australia, China, and Far East with very high level of nucleotide and amino acid sequence identities. Analysis of Seg-5 showed its grouping within the western topotype cluster, a phenomenon that is repeatedly being seen in isolates collected post-1982. Possibly, the western NS1 contributes to enhanced transmission of the virus. Analyses of serotype-determining segments (Seg-2 and Seg-6) have grouped IND2014/01 within serotype 16, with high level of sequence identity (99%) to the previous BTV-16 isolates within the eastern topotype, confirming its serotype.

Multiple BTV serotypes are currently circulating in the Indian subcontinent (5, 12, 13), thus potentially creating opportunities for the generation and circulation of novel reassortant viruses with unique characteristics. Hence, full-genome constellation analysis and sharing of genomic data are warranted to timely identify the newly emergent viruses.

### Accession number(s).

The complete genome sequence of BTV isolate IND2014/01 was deposited in GenBank under the accession numbers KX302634 to KX302643.
